# miR-142-5p in Bone Marrow-Derived Mesenchymal Stem Cells Promotes Osteoporosis Involving Targeting Adhesion Molecule VCAM-1 and Inhibiting Cell Migration

**DOI:** 10.1155/2018/3274641

**Published:** 2018-03-29

**Authors:** Zhaowei Teng, Xueguan Xie, Yun Zhu, Jianping Liu, Xingbo Hu, Qiang Na, Xiongwen Zhang, Guojun Wei, Shen Xu, Yugang Liu, Xiguang Zhang, Cory J. Xian

**Affiliations:** ^1^Department of Orthopedic Surgery, The People's Hospital of Yuxi City, The 6th Affiliated Hospital of Kunming Medical University, Yuxi, Yunnan 653100, China; ^2^Huai'an Second People's Hospital, The Affiliated Huai'an Hospital of Xuzhou Medical University, Huai'an, Jiangsu 223200, China; ^3^Health Screening Center, The People's Hospital of Yuxi City, The 6th Affiliated Hospital of Kunming Medical University, 21 Nieer Road, Yuxi, Yunnan 653100, China; ^4^Department of Orthopedics, The First People's Hospital of Kunming, Kunming, Yunnan 650011, China; ^5^Department of Orthopedics, The First Affiliated Hospital of Harbin Medical University, Harbin, Heilongjiang 150006, China; ^6^Department of Orthopedics, Affiliated Hospital of Hebei University of Engineering, Handan, Hebei 056002, China; ^7^Sansom Institute for Health Research, School of Pharmacy and Medical Sciences, University of South Australia, Adelaide, SA 5001, Australia

## Abstract

Osteoporosis is a systemic bone metabolic disease that is highly prevalent in the elderly population, particularly in postmenopausal women, which results in enhanced bone fragility and an increased susceptibility to fractures. However, the underlying molecular pathogenesis mechanisms still remain to be further elucidated. In this study, in a rat ovariectomy- (OVX-) induced postmenopausal osteoporosis model, aberrant expression of a microRNA miR-142-5p and vascular cell adhesion molecule 1 (VCAM-1) was found by RNA sequencing analysis and qRT-PCR. Using a dual-luciferase reporter assay, we found that miR-142-5p can bind to and decrease expression of VCAM-1 mRNA. Such reduction was prohibited when the miR-142-5p binding site in VCAM-1 3′UTR was deleted, and Western blotting analyses validated the fact that miR-142-5p inhibited the expression of VCAM-1 protein. Bone marrow-derived mesenchymal stem cells (BMMSCs) transfected with miR-142-5p showed a significantly decreased migration ability in a Transwell migration assay. Collectively, these data indicated the important role of miR-142-5p in osteoporosis development involving targeting VCAM-1 and inhibiting BMMSC migration.

## 1. Introduction

Postmenopausal osteoporosis (OPM) is the most common type of osteoporosis. As a result of the decrease in postmenopausal estrogen levels, bone microstructure deteriorates, bone mineral density (BMD) decreases, and bone fragility increases, with bone fractures often occurring in the severe cases [1, 2]. At present, the incidence of OPM in women over 50 years of age is higher than 50%, and the world incidence of OPM fractures has increased by 18% in five years, which seriously affects the health and quality of life of middle-aged and old women [3]. While bone marrow-derived mesenchymal stem cells (BMMSCs) can differentiate into osteoblasts and are known to play an important role in bone development, regeneration, and repair, as a leading causative event of the process leading to osteoporosis, the osteogenic differentiation of BMMSCs has been shown to be reduced [4, 5]. At present, the clinical treatments of osteoporosis mainly include hormone replacement, calcium supplementation, inhibition of bone resorption, and other methods. However, due to drug side effects, malabsorption, efficacy instability, poor patient compliance, and other reasons, the treatment effect is not ideal. More research is required to further understand the pathophysiology of OPM and to devise novel effective treatments.

MicroRNAs (miRNAs) are a class of noncoding single-stranded RNAs encoded by endogenous genes of about 22 nucleotides, typically associating with the 3′UTR region or coding region of the target gene mRNA sequence, which thus regulates protein expression of the target gene [6, 7]. For example, it has been reported that miR-142-5p is an important noncoding RNA targeting several genes depending on cellular microenvironments. Recent studies have shown that a variety of miRNAs play an important regulatory role in BMMSC cell differentiation [8–10]. However, miRNAs involved in regulating BMMSC osteogenic differentiation in osteoporosis are largely unknown. As a major adhesion protein, vascular cell adhesion molecule 1 (VCAM-1) has been found to be expressed at lower levels in some diseases that are characterized by a deficiency or dysfunction in mesenchymal stem cells, such as aplastic anemia. However, potential functions of miR-142-5p and VCAM-1 in osteoporosis have been hardly reported previously.

Thus, in this study, miRNA sequencing was conducted in BMMSCs isolated from a rat model of ovariectomy- (OVX-) induced OPM, from which miRNAs associated with the development of OPM were screened. After expression confirmation, miR-142-5p with a clear expression difference following OVX was selected for subsequent analyses to study how it regulates the behaviours and functions of BMMSCs.

## 2. Results

### 2.1. Significant Trabecular Bone Loss at Days 45 and 90 in a Rat Model of Total OVX-Induced Osteoporosis

**Figure 1:**
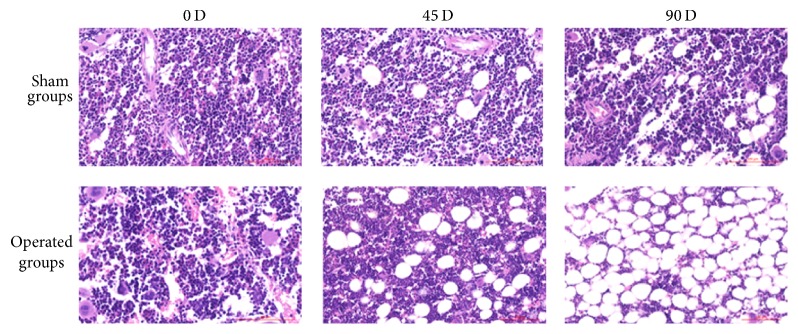
Representative images of H&E-stained sections of femurs (at the lower secondary spongiosa region) of rats at different time points (0, 45, or 90 days) after sham or OVX operation (×100).

A bilateral OVX-induced osteoporosis model was established in the current study according to a previous study [11], and the BMD was measured on days 0, 45, and 90 after OVX (Table 1). Significant differences in BMD between the sham and OVX groups at the 45th day and the 90th day (*p* < 0.05) suggest significant bone loss in rats at 45 and 90 days after ovariectomy. As a verification, our histology analyses of H&E-stained sections of femurs (at the lower secondary spongiosa region) of rats at different time points after sham or OVX operation confirmed our BMD findings. It can be seen that trabecular spacing significantly increased in OVX rats at day 45 when compared to day 0 or sham control, and the spacing increased further at the 90th day in OVX rats when compared to sham rats (Figure 1). These results reveal that OVX animals on days 45 and 90 showed significant symptoms of osteoporosis.

### 2.2. Significantly Increased Expression of miR-142-5p in BMMSCs Isolated from OVX-Induced Osteoporotic Rats

**Figure 2:**
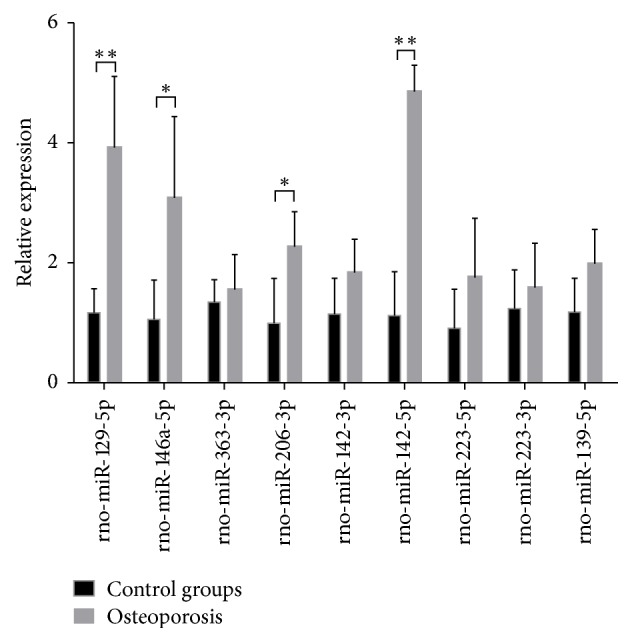
Relative expression of miRNAs (of >5-fold changes) as analysed by qPCR in BMMSCs isolated from sham control rats or OVX-induced osteoporosis rats (*n* = 5/group; ^*∗*^*p* < 0.05 and ^*∗∗*^*p* < 0.01).

BMMSCs were isolated from days 0, 45, and 90 after sham or OVX operation and miRNAs were sequenced. Among them, the miRNAs that had expression differences over 3 folds are tabulated (Table 2). Furthermore, for expression verification, BMMSCs were isolated from the sham group and OVX group of day 45, from which miRNAs were extracted and used in quantitative PCR analyses of those miRNAs with over 5-fold differences as revealed from the above sequencing analyses (Figure 2). As shown, there are significant differences in four miRNAs between the sham and OVX groups. Among them, *p* values of miR-129-5p and miR-142-5p were both less than 0.01, being 0.0012 and 0.000058, respectively. *p* values of miR-146a-5p and miR-206-3p were both less than 0.05, being 0.016 and 0.018, respectively. As miR-142-5p was found to have the lowest *p* value, it was selected to be studied further.

### 2.3. miR-142-5p Inhibited VCAM-1 Expression by Binding to the 3′UTR Region of VCAM-1

**Figure 3:**
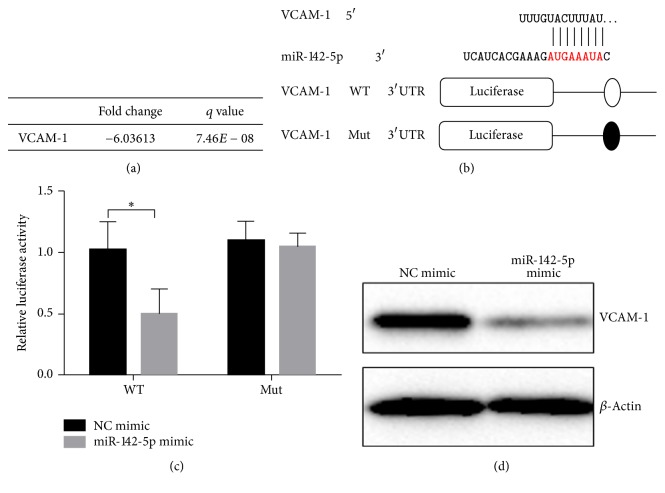
miR-142-5p can inhibit the expression of VCAM-1. (a) Results of VCAM-1 in BMMSCs transcriptome sequencing. (b) A schematic diagram of miR-142-5p paring with the 3′UTR area of VCAM-1, as well as the schematic diagram of the luciferase expression vector constructs, respectively, with wild-type (WT) and mutant (Mut) VCAM-1 sequences. (c) Test results of the dual-luciferase activity assay; *n* = 3 for each group. (d) Transfection with the miR-142-5p mimic, but not the negative control (NC) mimic, inhibits protein expression of VCAM-1 as revealed by Western blotting analyses. ^*∗*^*p* < 0.05.

By analysing miRWalk database to identify mRNA that binds to miR-142-5p, miR-142-5p was found to be able to bind to the 3′UTR region of VCAM-1. Consistently, after analysing the transcriptome sequencing data of BMMSCs from our previous rat OVS osteoporotic model, the expression of VCAM-1 in BMMSCs was significantly decreased in the OVX osteoporotic rats (Figure 3(a)). The binding sequence of miR-142-5p to VCAM-1 in the 3′UTR region is shown in Figure 3(b).

To confirm that miR-142-5p indeed binds to VCAM-1 3′UTR region and regulates VCAM-1 expression, the 3′UTR region of VCAM-1 was extracted and the 8 nucleotides of the binding domain were knocked down prior to being constructed into the luciferase expression vector. The effect of miR-142-5p on luciferase expression was detected by the dual-luciferase assay (Figure 3(c)). The results revealed that when VCAM-1 sequence was intact, miR-142-5p significantly reduced the luciferase activity in the 3′UTR region of VCAM-1. However, when the sequence of binding to miR-142-5p in the 3′UTR region of VCAM-1 was knocked down, the luciferase activity was not affected by miR-142-5p. Furthermore, cells transfected with miR-142-5p mimic, but not the negative control mimic, were shown to have a much lower VCAM-1 protein expression as revealed via Western blotting analyses (Figure 3(d)). These results showed that miR-142-5p can significantly inhibit the expression of VCAM-1 and that miR-142-5p inhibits the expression of VCAM-1 by binding to the AUGAAAUA sequence of the 3′UTR region of VCAM-1.

### 2.4. miR-142-5p Inhibited Migration of BMMSCs

**Figure 4:**
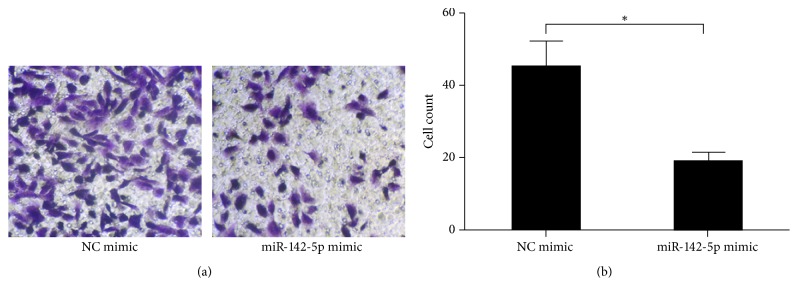
miR-142-5p mimic, but not the negative control (NC) mimic, inhibited the migration ability of bone marrow-derived mesenchymal stem cells. (a) Representative images of migrated cells in a Transwell cell migration assay. (b) Statistical analyses of three Transwell migration experiments. ^*∗*^*p* < 0.05.

The migration ability of BMMSCs is related to the level of osteogenesis, and impaired osteogenesis is an important part of the development of osteoporosis. Therefore, to study whether miR-142-5p affects the migration of BMMSCs, the BMMSCs were isolated from sham and OVX groups and their abilities to migrate in a Transwell migration assay were examined following transfection with the miR-142-5p mimic or negative control mimic (Figure 4). The migration ability of BMMSCs transfected with miR-142-5p mimic was significantly decreased, while there were no significant differences in the group transfected with negative control mimic. The results showed that miR-142-5p could inhibit the migration of BMMSCs by inhibiting the expression of VCAM-1.

## 3. Discussion

Estrogen deficiency is one of the major risk factors of osteoporosis. Osteoporosis results from imbalanced and negative bone remodelling when osteogenesis (carried out by bone forming cells osteoblasts) is less than bone resorption (carried out by resorptive cells osteoclasts). BMMSCs are precursors of osteoblasts, and when BMMSCs fail to migrate and differentiate to osteoblasts in areas needed, decreased osteogenesis would ensure causing osteoporosis [12]. However, how the migration of BMMSCs is regulated in osteoporosis remains unclear.

It has been reported that miRNAs regulate gene expression in physiological and pathophysiologic process [13]. Although dozens of miRNAs have been suggested to play roles in osteoporosis [14], their regulatory roles in osteoporosis are unclear. In this present study, using an OVX osteoporosis model in rats, many miRNAs were found to have significant changes in expression, and, particularly, miR-142-5p was found to have the most significant induction in BMMSCs of rats with OVX-induced osteoporosis.

Previously, miR-142-5p has been reported to be able to regulate several genes depending on cellular microenvironments, and its aberrant expression has been detected in various disorders. Song and Kim reported that miR-142-5p levels correlated to A*β*42 expression [15]. Sharma reviewed that miR-142-5p can regulate inflammation and immune responses [16]. miR-142-5p was also reported to play roles in colon cancer and squamous cell carcinoma [17, 18]. However, the target gene(s) and molecular mechanism of miR-142-5p action in osteoporosis are not clear yet.

The current study has investigated the relationship between miR-142-5p and VCAM-1 in the osteoporosis setting. VCAM-l is the 100 kDa or 110 kDa transmembrane glycoprotein named CD106, a high-affinity adhesion molecule, which is commonly expressed in endothelial cells, bone marrow stromal cells, smooth muscle cells, macrophages, and dendritic cells [19]. VCAM-1 is an adhesion protein important in cell-cell recognition and it plays multiple roles in inflammation, cell differentiation and development of organs, immune responses, lymphocyte homing and recycling, and malignant metastasis [20]. VCAM-1 in the immune cells is known to contribute to their activation and migration [21]. Furthermore, VCAM-1 is expressed at lower levels in some diseases characterized with mesenchymal stem cell deficiency or dysfunction, such as aplastic anemia. In the current study, based on our previous transcriptome sequencing analysis, miR-142-5p was proven to bind 3′UTR in VCAM-1 gene and inhibit BMMSC migration by downregulating VCAM-1 expression. Due to the important role of VCAM-1 in migration of BMMSCs and the critical role of BMMSCs migration to site of bone formation prior to their osteogenic commitment for new bone formation during physiological bone remodelling and bone fracture healing, the findings in the current study indicated the important role of miR-142-5p in osteoporosis development involving targeting VCAM-1 and inhibiting BMMSC migration.

## 4. Materials and Methods

### 4.1. Animals and OVX-Induced Osteoporosis Model

All procedures were approved by the Animal Care and Use Committee of Kunming Medical University. 30 ten-week-old healthy specific-pathogen-free (SPF) female Sprague-Dawley (SD) rats were purchased from Vital River (Beijing, China). All rats were preserved under standard housing laboratory conditions. After one week of adaptation to the diet and the new environment, the rats were randomly divided into 2 groups: ovariectomized group (OVX, *n* = 15) and sham-operated group (sham, *n* = 15). At 12 weeks of age, OVX group underwent OVX (removal of bilateral ovaries) after being anesthetized via i.p. injection of pentobarbital sodium in PBS at 30 mg/kg as we described and according to the classic protocol [11, 22, 23]. For the sham operation group, the same volume of fat pad was taken out instead of the bilateral ovaries.

### 4.2. Specimen Collection, BMD Measurement, and Histology Staining

The rats were sacrificed with carbon dioxide overdose at 45 days and 90 days after operation. The BMDs of left femurs were measured using dual-energy X-ray absorptiometry (DEXA) (Prodigy, Lunar, GE Healthcare, Madison, WI) (mg/cm^2^). Femoral specimens were fixed with 4% paraformaldehyde, decalcified (10% EDTA-2Na, 0.1 M Tris buffer, pH 7.3), and embedded in paraffin sections. Sections of 4 *μ*m were cut, stained by H&E, and photographed under light microscope.

### 4.3. Isolation and Cultivation of BMMSCs

For isolation of BMMSCs, rats were sacrificed by cervical dislocation and soaked in 75% ethanol for 10 min, and then femurs and tibias were dissected under sterile condition and washed with PBS three times. After removing epiphyses, bones were flushed with ECM medium (ScienCell, Carlsbad, CA) supplemented with 10% fetal bovine serum (Gibco, Grand Island, NY) and 100 U/ml penicillin and streptomycin (Sigma-Aldrich, St. Louis, MO). The isolated bone marrow samples were made into single cell suspension and centrifuged at 1000 rpm/min for 5 min, and after the supernatant was discarded, the cells were resuspended in a concentration of 1 × 10^9^ L^−1^ in a 25 cm^2^ plastic culture flask for incubation at 37°C in a 5% CO_2_ incubator. During the primary stromal cell culture, the medium was replaced after 48 h, and the fresh medium was replaced every 3 days. For passage culture, cells were trypsinised and then subcultured at 1 : 2 ratio.

### 4.4. MiRNA Sequencing

For miRNA sequencing purpose, after BMMSCs were cultured for 10 days, 1 mL TRIzol (Invitrogen, Carlsbad, CA) was added to 1 × 10^7^ cells of BBMSCs. Then, the samples were sent to Novogene (Beijing, China) for sequencing and data analysis.

### 4.5. Quantitative PCR (qPCR)

The intracellular miRNA was extracted by miRNA extraction kit (DP501, Tiangen, Beijing, China), and reverse transcription was performed using RT-PCR kit (KR108, Tiangen). For quantitative PCR, ABI7500 real-time PCR system (Applied Biosystems, Beijing, China) was used to perform quantitative experiments using SYBR Green kit (FP201, Tiangen). The expression levels of miR-142-5p and other miRNAs were normalized by U6. The primer sequences are listed in Table 3.

### 4.6. Western Blotting Analyses

Cells were lysed in RIPA buffer plus protease inhibitors (Roche, South San Francisco, CA). Equal amounts of proteins were loaded and separated on 10% SDS-PAGE and then transferred to a nitrocellulose membrane (BD Biosciences, San Jose, CA), blocked by incubation with 5% fat-free milk in TBST buffer (150 mM NaCl, 50 mM Tris-HCl, 0.5% Tween 20, pH 7.6) at room temperature for 1 h. The membranes were incubated with primary antibodies against vascular cell adhesion molecule 1 (VCAM-1) (ab134047, Abcam, Cambridge, UK) or against the internal control *β*-actin (Sigma-Aldrich) at 4°C overnight and then with horseradish peroxide-conjugated secondary antibodies at room temperature for 1 h, prior to being developed with ECL reagent (Thermo Scientific, Waltham, MA) as described [24].

### 4.7. Dual-Luciferase Assay

The 3′UTR region of VCAM-1 was amplified by PCR and constructed into pGL3 vector. The upstream primer was 5′-CTAGCTAGCCGTCTGAACTGTTGGAG-3′ with XhoI as the cleavage site, and the downstream primer was 5′-CCCAAGCTTCTTAATCAGTATACCATC-3′ with HindIII as the cleavage site. The mutant primers were as follows: upstream: 5′-TTGAAACTTCAGCCTAA-3′, downstream: 5′-AAACAAAAAGGTGACAC-3′. Using transfection reagent Lipofectamine 2000 (Invitrogen, Carlsbad, CA), HEK293T cells were cotransfected with pGL3-VCAM-1-3′UTR-WT (with wild-type VCAM-1 sequence) or pGL3-VCAM-1-3′UTR-MUT (with 8 nucleotides of the VCAM-1 3′UTR region being mutated) (see below) and pRL and miR-142-5p mimic or negative control (NC) mimic (Genscript, Beijing, China). The cells were harvested after 48 hours to detect dual-luciferase activities using dual-luciferase assay kit (Promega, Madison, WI) as described [25].

### 4.8. Statistical Analyses

GraphPad Prism 7.0 was used to evaluate data. The experimental data was expressed as *χ* ± *s*, and the data was analysed by *t*-test or single-factor analysis of variance. All experiments were repeated at least three times to show representative results. *p* < 0.05 was considered statistically significant.

## 5. Conclusions

miR-142-5p has been proven in the current study to be the most significantly induced miRNA in BMMSCs and bind to 3′UTR of VCAM-1 to inhibit VCAM-1 expression in OVX-induced osteoporosis in rats. This study has also shown that downregulated VCAM-1 inhibits migration of BMMSCs in osteoporosis. Thus, this work has presented evidence for a potentially new target for diagnosis, prevention, and therapy of osteoporosis.

## Figures and Tables

**Table 1:** Results of the bone mineral density in rats of the sham or OVX groups at different time points after operation.

	0 days (mg/cm^2^)	45 days (mg/cm^2^)	90 days (mg/cm^2^)
Sham	174.18 ± 18.66	169.87 ± 15.82	162.29 ± 19.83
OVX	168.78 ± 23.89	145.64 ± 17.80	114.94 ± 19.92
*p* value	>0.05	<0.05	<0.05

**Table 2:** The list of miRNAs that have at least 3-fold changes in the gene sequencing analyses.

miRNA	Fold Change	*q* value
rno-miR-129-5p	29.90399529	1.99*E* − 228
rno-miR-146a-5p	8.6247	0
rno-miR-363-3p	7.6276	0.00072763
rno-miR-206-3p	7.0579	1.07*E* − 32
rno-miR-142-3p	6.9643	8.18*E* − 18
rno-miR-142-5p	6.5649	5.45*E* − 38
rno-miR-223-5p	6.3947	8.10*E* − 05
rno-miR-223-3p	6.1517	1.12*E* − 10
rno-miR-139-5p	5.829	6.40*E* − 13
rno-miR-6321	4.091	0.00042924
rno-miR-184	3.5614	2.51*E* − 05
rno-miR-325-3p	3.4431	0.0084137
rno-miR-455-5p	3.3579	2.04*E* − 134
rno-miR-10b-5p	3.3451	0
rno-miR-204-5p	3.2485	1.25*E* − 42
rno-miR-708-3p	3.2432	9.74*E* − 14
rno-miR-29c-5p	3.1197	6.74*E* − 06
rno-miR-140-3p	−3.0982	0
rno-miR-541-5p	−3.1068	1.72*E* − 06
rno-miR-582-5p	−3.1309	0.00023355
rno-miR-379-5p	−3.438	5.02*E* − 13
rno-miR-127-3p	−4.1729	2.29*E* − 30
rno-miR-129-1-3p	−4.7145	1.44*E* − 19
rno-miR-129-2-3p	−4.7145	1.44*E* − 19

**Table 3:** The sequences of reverse transcriptional primers and qPCR primers used in miRNA quantitative PCR.

Primers	Sequences (5′-3′)
rno-miR-129-5p reverse transcription sequence	GTCGTATCCAGTGCAGGGTCCGAGGTATTCGCACTGGATACGACGCAAGC
rno-miR-129-5p qPCR upstream primer	GCGGCTTTTTGCGGTCTGG
rno-miR-146a-5p reverse transcription sequence	GTCGTATCCAGTGCAGGGTCCGAGGTATTCGCACTGGATACGACAACCCA
rno-miR-146a-5p qPCR upstream primer	GCGGTGAGAACTGAATTCCA
rno-miR-363-3p reverse transcription sequence	GTCGTATCCAGTGCAGGGTCCGAGGTATTCGCACTGGATACGACACAGAT
rno-miR-363-3p qPCR upstream primer	GCGGAATTGCACGGTATCC
rno-miR-206-3p reverse transcription sequence	GTCGTATCCAGTGCAGGGTCCGAGGTATTCGCACTGGATACGACCCACAC
rno-miR-206-3p qPCR upstream primer	GCGGTGGAATGTAAGGAAGT
rno-miR-142-3p reverse transcription sequence	GTCGTATCCAGTGCAGGGTCCGAGGTATTCGCACTGGATACGACTCCATA
rno-miR-142-3p qPCR upstream primer	GCGGTGTAGTGTTCCTACTT
rno-miR-142-5p reverse transcription sequence	GTCGTATCCAGTGCAGGGTCCGAGGTATTCGCACTGGATACGACAGTAGT
rno-miR-142-5p qPCR upstream primer	GCGGCATAAAGTAGAAAGC
rno-miR-223-5p reverse transcription sequence	GTCGTATCCAGTGCAGGGTCCGAGGTATTCGCACTGGATACGACCAACTC
rno-miR-223-5p qPCR upstream primer	GCGGCGTGTATTTGACAAGCT
rno-miR-223-3p reverse transcription sequence	GTCGTATCCAGTGCAGGGTCCGAGGTATTCGCACTGGATACGACGGGGTA
rno-miR-223-3p qPCR upstream primer	GCGGTGTCAGTTTGTCAAA
rno-miR-139-5p reverse transcription sequence	GTCGTATCCAGTGCAGGGTCCGAGGTATTCGCACTGGATACGACCTGGAG
rno-miR-139-5p qPCR upstream prime	GCGGTCTACAGTGCACGTGT
qPCR common downstream sequence	GTGCAGGGTCCGAGGT
